# Highly Efficient Four-Rod Pumping Approach for the Most Stable Solar Laser Emission

**DOI:** 10.3390/mi13101670

**Published:** 2022-10-04

**Authors:** Miguel Catela, Dawei Liang, Cláudia R. Vistas, Dário Garcia, Hugo Costa, Bruno D. Tibúrcio, Joana Almeida

**Affiliations:** Center of Physics and Technological Research (CEFITEC), Department of Physics, Faculty of Science and Technology, NOVA University of Lisbon, 2829-516 Caparica, Portugal

**Keywords:** solar-pumped laser, multi-rod, solar tracking error, numerical analysis

## Abstract

We report a significant numerical improvement in multi-rod laser efficiency, with an enhanced solar tracking error compensation capacity for a heliostat-parabolic system. The solar laser head was composed of a fused silica conical lens and a single conical pump cavity ensuring multiple passes through four 4.55 mm diameter, 15 mm length Nd:YAG rods. 0.76° tracking error width at 10% laser power loss, and total multimode laser power variation of 0.05% at ±0.1° solar tracking error and 0.30% at ±0.2° solar tracking error were numerically calculated, being 1.27, 74.80 and 21.63 times, respectively, more than the experimental record in solar tracking error compensation capacity attained with a dual-rod side-pumping horizontal prototype pumped by the same heliostat-parabolic system. Additionally, the end-side-pumping configuration of the four-rod solar laser-enabled 43.7 W total multimode solar laser power, leading to 24.7 W/m^2^ collection efficiency and 2.6% solar-to-laser power conversion efficiency, being 1.75 and 1.44 times, respectively, more than that experimentally obtained from the dual-rod side-pumping prototype. The significant improvement in solar tracking error compensation capacity with a highly efficient end-side-pumping configuration is meaningful because it reduces the cost of high-precision trackers for solar laser applications.

## 1. Introduction

The direct conversion of natural sunlight into laser light may be considered as one of the most promising technologies in renewable energy research, providing a cost-effective production of coherent optical radiation that could lead to numerous environmental and economic benefits [1,2]. As a future emerging technology for space applications, direct solar laser pumping offers unique simplicity in not requiring any artificial electrical power generation and power conditioning equipment. Among the potential space applications are atmospheric and oceanic sensing, laser power beaming, deep space communications, laser propulsion and removal of orbital space debris [3,4,5,6]. On Earth, solar laser can be used in high-temperature materials processing [7].

Solar laser emission was first reported in 1963 by Kiss et al. [8], not long after the invention of laser [9]. Gas, liquid and solid laser media have been studied [10,11,12]; however, solid-state media’s inherent high energy density, compactness, and potential for efficient solar-to-laser power conversion have made them widely used [12,13,14,15,16,17,18,19,20,21,22,23,24,25,26]. Particularly, Nd:YAG material has been proven to be quite effective in solar-pumped lasers due to its suitability for solar pumping of high intensity as a result of the spectroscopic properties of the dopant [22], and good thermomechanical properties, making it durable and resilient to thermal fractures [23], along with its availability and relatively low cost. In the first Nd:YAG solar laser systems, efforts were made to increase the laser output power from 1.0 W in 1966 to 60.0 W in 1988 [12,13,14]. In order to compare the solar laser output performance obtained from different primary concentrators, research teams have been using the solar laser collection efficiency figure-of-merit [15,16,17,18,19,20,21,22,23,24,25,26]. It provides a very straightforward way to find out the amount of solar laser power produced from each square meter of collector area, although it may be misleading since this parameter is dependent on the solar irradiance, which can vary largely with geographical location. Solar-to-laser conversion efficiency is defined as the percentage of laser power produced from the total disposable input solar pump power. A clear advantage of this parameter lies in its independence from the solar irradiance. Solar laser researchers continued to improve the collection and solar-to-laser conversion efficiencies using either highly focusing parabolic mirrors, or lightweight, low-cost, and easily available Fresnel lenses. In 2011, 19.3 W/m^2^ solar laser collection efficiency and 2.2% solar-to-laser conversion efficiency were achieved by exciting a 4.0 mm diameter, 25 mm length Nd:YAG rod with a Fresnel lens with 0.64 m^2^ collection area [17]. A 30.0 W/m^2^ solar laser collection efficiency and a 3.3% solar-to-laser conversion efficiency were attained by pumping a 6.0 mm diameter, 100 mm length Nd:YAG rod through a large Fresnel lens with 4 m^2^ collection area in 2012 [18]. In 2017, 37.2 W of continuous-wave (CW) multimode 1064 nm solar laser power was produced from a 4.0 mm diameter, 35 mm length Nd:YAG rod through a parabolic mirror with 1.18 m^2^ collection area, resulting in 31.5 W/m^2^ solar laser collection efficiency and 3.1% solar-to-laser conversion efficiency [25]. In 2018, a 4.5 mm diameter, 35 mm length Cr:Nd:YAG ceramic rod was efficiently pumped through a parabolic mirror with 1.0 m^2^ effective collection area, leading to the state-of-the-art solar laser collection efficiency of 32.5 W/m^2^ and solar-to-laser conversion efficiency of 3.7% [26].

All the above-mentioned achievements were accomplished with solar laser schemes based on single-laser/single-beam concept. However, the single-spot technology can demand days and even weeks for processing materials [27]. Research has produced a trend towards the deployment of multiple laser beams, each optimized to perform a facet of the overall process [28]. By using a layout of many lasers, a multi-laser/multi-beam arrangement can decrease the processing time. Still, due to the size of the lasers and its beam delivery systems, this approach is complex and limited in the maximum number of deployable laser sources [29]. On the other hand, high compactness and simplicity can be achieved with a single-laser/multi-beam concept based on light-splitting technologies [30]. Nevertheless, laser output power and its beam quality are strongly influenced by the thermal lensing and thermal stress problems of the single-laser source. To avoid these limitations, solar laser systems based on a multi-rod/multi-beam concept from a single solar laser head have been studied [31,32,33,34,35,36]. This is significant for solar laser research, since solar pump rays not completely absorbed by one of the rods can be further absorbed by other rods, leading to more passages through the active media. This increases the path length of the rays inside the rods and hence the amount of energy absorbed, while avoiding the serious thermal fracture, thermal lensing and thermal stress issues associated with classical single-thick-rod solar lasers [37]. Almeida et al. proposed a four-rod pumping scheme for a 2.0 m diameter parabolic mirror [31]. A quadrangular pyramidal reflector was used to redirect the focused solar rays towards four laser heads, each one consisting of a double-stage biconical lens/conical pump cavity and a small diameter Nd:YAG rod. Total multimode solar laser power of 59.0 W was obtained in the numerical analysis, leading to 19.2 W/m^2^ collection efficiency and 2.0% solar-to-laser conversion efficiency. In 2020, the first simultaneous emission of three 1064 nm CW solar laser beams was reported by end-side-pumping three 3.0 mm diameter, 25 mm length Nd:YAG rods within a single conical pump cavity with a heliostat-parabolic system. Substantial improvement in the laser rods’ thermal performance was achieved, when compared to that of the single rod pumped within the same pump cavity. Total multimode solar laser power of 18.3 W was measured at an effective collection area of 1 m^2^, resulting in 18.3 W/m^2^ collection efficiency and 2.2% solar-to-laser conversion efficiency [32].

Solar tracking systems (STS) have an important role in the development and efficiency of solar concentration applications [38]. Since the first STS, introduced in 1962 [39], many other researchers have investigated solar trackers in order to improve their solar collection efficiency [40,41]. STS can have simple operation principles, without mechanical parts or sensing units to help them track the Sun [42]. However, those systems have lower accuracy and energy gain compared to their more complex counterparts [43]. A solar concentrator with a medium or high concentration ratio needs to be orientated correctly by an accurate STS to avoid losing the sunrays from the receiver. Consequently, it becomes necessary to follow the motion of the Sun in both altitude and azimuth directions [44]. A single-axis STS can attain a direct tracking error smaller than ±0.40° [45], whereas a more complex STS, that makes use of a dual-axis mechanism, can directly track the Sun with an error of ±0.15° [46]. Dual-rod side-pumping approaches have been numerically and experimentally studied for stable solar laser emission with a heliostat-parabolic system, achieving high tracking error tolerances regarding the limiting collection efficiency of 14.1 W/m^2^ and solar-to-laser conversion efficiency of 1.8% [34,36]. In fact, the current record in Nd:YAG solar laser collection efficiency by a side-pumping configuration is only 17.60 W/m^2^ [47]. Despite single-rod end-side-pumping configurations being able to attain far superior efficiency [25,26], the concentrated solar power is directly focused onto the end face of the laser rod, and any slight solar tracking error in either azimuth or altitude axis can prevent its stable emission, causing a significant reduction in or even the extinction of solar laser output.

A four-rod/four-beam single solar laser head is proposed here, in order to improve the solar laser efficiency and solar tracking error compensation capacity with end-side-pumping configuration, while reducing significantly the thermal lensing and thermal stress problems that have affected single-rod solar lasers [48,49]. A prototype was designed through ZEMAX^®^ (Kirkland, WA, USA) and laser cavity analysis and design (LASCAD^®^, Munich, Germany) software. A heliostat-parabolic system with 1.767 m^2^ collection area was used to collect and concentrate the solar radiation. A fused silica conical lens further concentrated the solar radiation onto the four Nd:YAG rods, within a single conical pump cavity. The highly concentrated radiation was distributed to pump several rods simultaneously, achieving very effective rod cooling. The maximum total multimode solar laser power of 51.2 W (4 × 12.8 W) was numerically calculated for the four rods with 3.00 mm diameter and 20 mm length, leading to a 29.0 W/m^2^ collection efficiency and a 3.1% solar-to-laser power conversion efficiency, being 1.58 and 1.41 times, respectively, more than the previous multi-rod experimental records in multimode regime with the same solar facility [32]. Moreover, by using four 4.55 mm diameter, 15 mm length Nd:YAG rods, the solar tracking error compensation capacity was significantly improved. A 0.76° tracking error width at 10% laser power loss, and total multimode laser power variation of 0.05% at ±0.1° solar tracking error and 0.30% at ±0.2° solar tracking error were numerically calculated, being 1.27, 74.80 and 21.63 times, respectively, more than the experimental record in solar tracking error compensation capacity obtained by using the dual-rod side-pumping horizontal prototype with the same heliostat-parabolic system [36]. In addition to the greater stability, the end-side-pumping four-rod solar laser head concept enabled 1.75 and 1.44 times more collection and solar-to-laser power conversion efficiencies than that of the experimentally obtained from the dual-rod side-pumping prototype [36].

## 2. Description of the Four-Rod Nd:YAG Solar Laser Head for a Heliostat-Parabolic System

### 2.1. Solar Energy Collection and Concentration System

**Figure 1:**
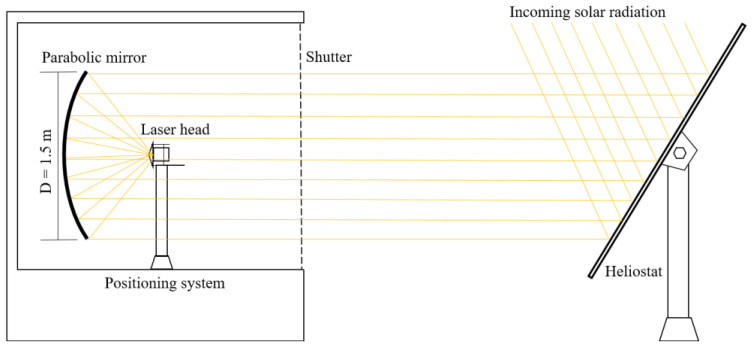
Schematics of the NOVA heliostat-parabolic system for pumping the four-rod Nd:YAG solar laser head.

As shown in Figure 1, the NOVA heliostat-parabolic system was composed of two large plane mirrors mounted on a two-axis heliostat that redirected the incoming solar radiation towards a stationary parabolic mirror with 1.5 m diameter, 60° rim angle and 660 mm focal length. A maximum effective collection area of 1.767 m^2^ was considered. All the mirrors were back-surface silver-coated. The low iron impurities within the glass substrates of the plane mirrors with 4 mm thickness allow around 93.5% of the incoming radiation to be reflected, whereas high iron impurities and large mirror thickness of 10 mm limits the reflectance of the primary parabolic mirror to about 80%. A total of 75% of incoming solar radiation was hence effectively concentrated. Assuming a maximum solar irradiance of 950 W/m^2^ in clear sunny days, the NOVA parabolic mirror focuses about 1250 W solar power into a highly concentrated pump light spot with near-Gaussian distribution with 8 mm full width at half maximum.

### 2.2. Four-Rod Single Solar Laser Head Design

**Figure 2:**
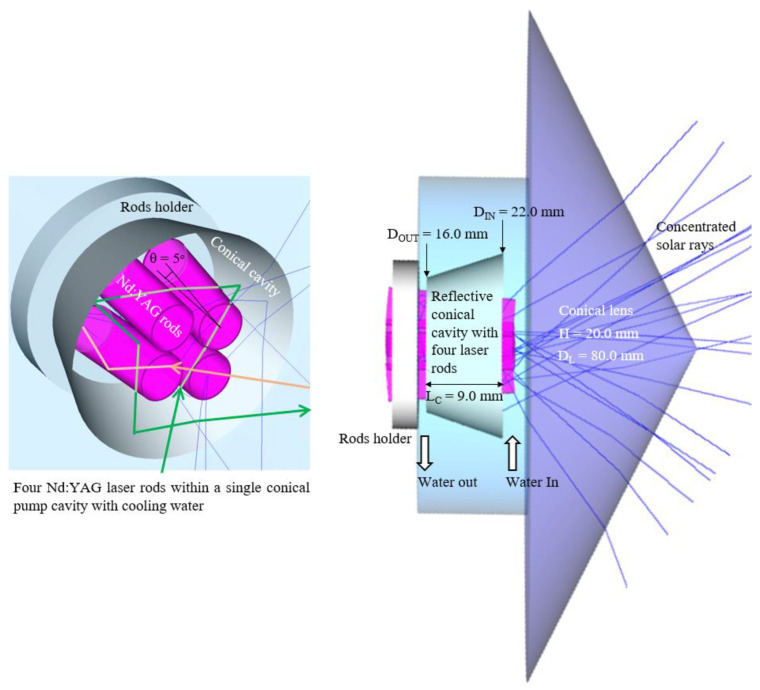
Design of the four-rod Nd:YAG laser head, composed of the fused silica conical lens, the conical pump cavity and the Nd:YAG rods, cooled by water. For end-pumping, the solar pump ray (orange ray) was directly focused onto the end face of one of the Nd:YAG rods, whereas for side-pumping, the solar pump ray (green ray) was redirected to the lateral surface of one of the laser rods through the conical cavity.

The single laser head was composed of the fused silica conical lens, the conical pump cavity, and the four Nd:YAG laser rods, as shown in Figure 2. Fused silica is an optimal material for solar laser pumping due to its low thermal expansion coefficient, resistance to scratching and thermal shock, and transparency over the visible spectrum broadbands. The conical shaped fused silica lens, with an output face diameter D_L_ = 80 mm and height H = 20 mm, efficiently coupled the concentrated solar radiation from the primary parabolic mirror into the four laser rods within the single conical pump cavity with 95% reflectivity.

As shown in Figure 2, for end-pumping (orange ray), one part of the concentrated radiation was directly focused onto the end face of the Nd:YAG rods through the conical lens, traveling within the rod through total internal reflection due to the refractive index differences between the water (*n_water_* ≈ 1.33) and the active medium (*n_Nd:YAG_* ≈ 1.82). For side-pumping (green ray), the rays entered the lateral surface of the rod with the help the conical reflector with input/output diameters D_IN_ = 22 mm/D_OUT_ = 16 mm and length L_C_ = 9 mm. An efficient multi-pass side-pump to the four Nd:YAG rods was hence ensured while permitting the energy sharing between the rods. Multiple passes can be achieved by a single ray zigzagging into the four crystals through the conical pump cavity, which increases the optical path length of the ray inside the rods and hence the amount of energy absorbed. Additionally, the concentrated solar rays that did not directly hit any of the rods are redirected by the reflective conical cavity towards one of the rods so that absorption is accomplished.

The four Nd:YAG rods, the pump cavity and the output facet of the conical lens were all actively cooled by circulating water. The constant flow of water was required for extracting the heat generated by the active medium in constant pumping, which helps in reducing both the thermal lensing and stress issue of the rod, thus increasing the lasing output. Water is an excellent coolant for solar lasers since it has a high thermal conductivity, specific heat, and low viscosity. Besides, since the refractive index of water is lower than that of fused silica (*n_silica_* ≈ 1.46), the direct contact of the conical lens output end with water ensures the efficient light coupling into the rods. As shown in Figure 2, the four Nd:YAG laser rods were evenly mounted on the rod holder in rotational symmetry, tilted θ = 5° in relation to the central axis, so as to create enough fixing spaces between the neighboring rods.

## 3. Numerical Modeling of the Four-Rod Solar Laser System through ZEMAX^®^ and LASCAD^®^ Software

Similar to our previous solar laser schemes [19,20,21,22,23,24,25,26,31,32,33,34,35,36], all the above-mentioned design parameters of the four-rod scheme were firstly optimized by non-sequential ray-tracing ZEMAX*^®^* software to achieve the maximum absorbed pump power for each laser rod, while ensuring an efficient redistribution of pump light to each one. The direct standard solar spectrum for one-and-a-half air mass (AM1.5 D) was used as the reference data for consulting the spectral irradiance (W m^−2^ nm^−1^) at each wavelength of the solar spectrum [50]. The terrestrial solar irradiance (*I*) of 950 W/m^2^ was considered in ZEMAX^®^. The effective pump power of the light source took into account the overlap efficiency (*η_OVP_*) of 16% between the absorption spectrum of Nd:YAG and the solar emission spectrum [51]. A source power (*P_Source_*) of about 269 W was calculated by equation 1 for a collection area *A* = 1.767 m^2^.

(1) $$P_{Source}=I\cdot A\cdot \eta_{OVP}$$

The Nd:YAG crystal offers advantages over other laser materials such as good thermal conductivity (14 W m^−1^ K^−1^), high quantum efficiency and tensile strength (200 N/mm^2^]) [23]. Spectral variation such as reflectance and transmission through the heliostat, parabolic mirror and the conical cavity were, respectively, programmed. The transmission spectrum and its wavelength-dependent refractive indexes of both fused silica and water were used from the glass catalog data within ZEMAX^®^. For the 1.0% Nd:YAG laser medium, about 22 absorption peak wavelengths were defined in ZEMAX^®^ numerical data. The relative weight of these wavelengths over the entire solar spectrum could be consulted from the standard solar spectra of AM1.5 D for the ZEMAX^®^ source.

**Figure 3:**
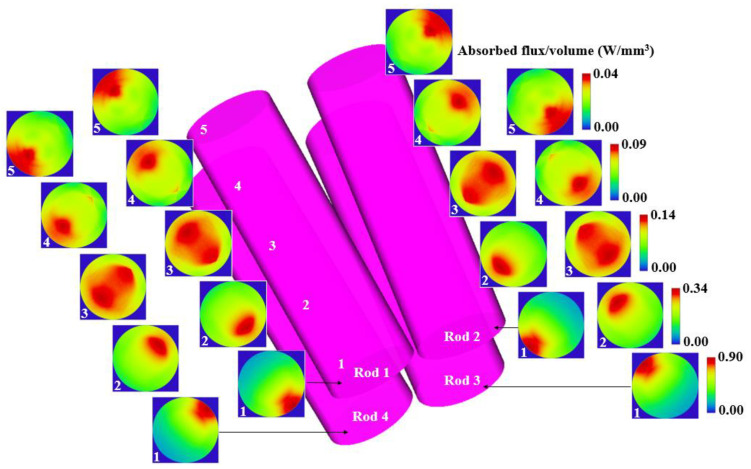
Absorbed pump-flux distributions along five transversal cross-sections (1,2,3,4 and 5) of the four Nd:YAG rods.

The pump flux distributions along five transversal cross-sections of the four Nd:YAG rods are given in Figure 3. Red color means near maximum solar energy absorption, whereas blue means little or no absorption. As expected, the four rods showed an identical absorbed pump profile with rotational symmetry around the center of the pump cavity. Therefore, the same absorbed pump power for the four rods was calculated. During ray-tracing, Nd:YAG rod was divided into 75,000 voxels, the path length of the rays through each voxel was found and, based on these values and on the effective absorption coefficient of the 1.0 at.% Nd:YAG material, the absorbed solar pump power within each laser rod was calculated.

**Figure 4:**
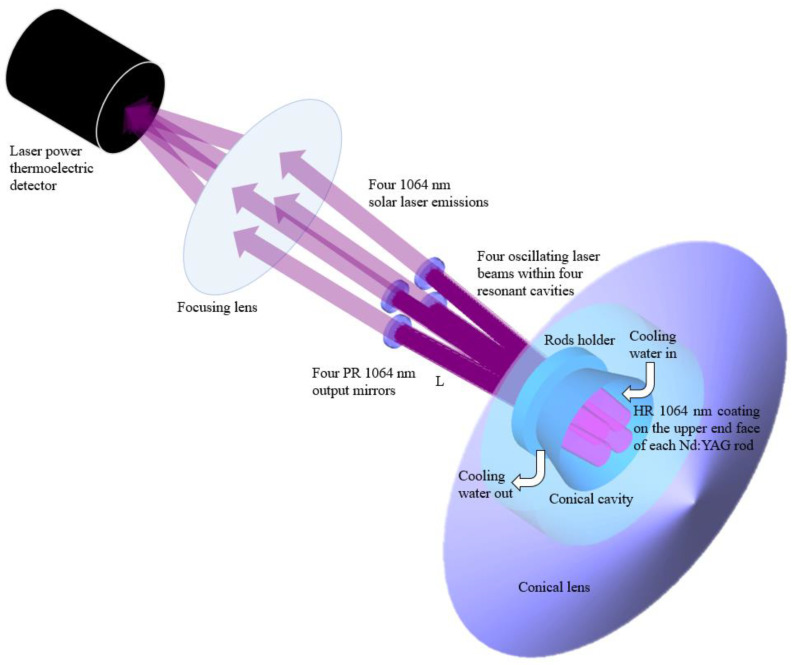
Solar-power-to-four-laser-beam conversion approach. Four PR 1064 nm laser output couplers were positioned near of their respective four Nd:YAG rods for the efficient extraction of four output laser beams.

The absorbed power in cubic matrix structure from the ZEMAX^®^ analysis was then imported into the LASCAD^®^ software to optimize the solar laser output performance. The material data of 1.0 at.% Nd:YAG crystal with a stimulated emission cross-section of 2.8 × 10^−19^ cm^2^, a fluorescence lifetime of 230 µs and an absorption/scattering loss of 0.002 cm^−1^ were set in LASCAD^®^. The mean absorbed and intensity-weighted solar pump wavelength of 660 nm was included in the analysis [23]. As represented in Figure 4, each optical resonator was comprised of two opposing mirrors: the high reflection (HR) coating of 99.98% on the upper end face of each Nd:YAG rod and the partial reflection (PR) output mirror, with reflectivity varying between 90% and 99% at the laser emission wavelength (1064 nm) in the present design. *L* represents the separation length between the output end face anti-reflection (AR 1064 nm) coatings of each Nd:YAG rod and the respective PR 1064 nm output mirror. It is a key parameter for the efficient extraction of laser power due to the power-dependent thermal lensing of the laser material.

## 4. Numerical Analysis of the Four-Rod Solar Laser Approach

### 4.1. Advances in Multimode Solar Laser Performance

**Figure 5:**
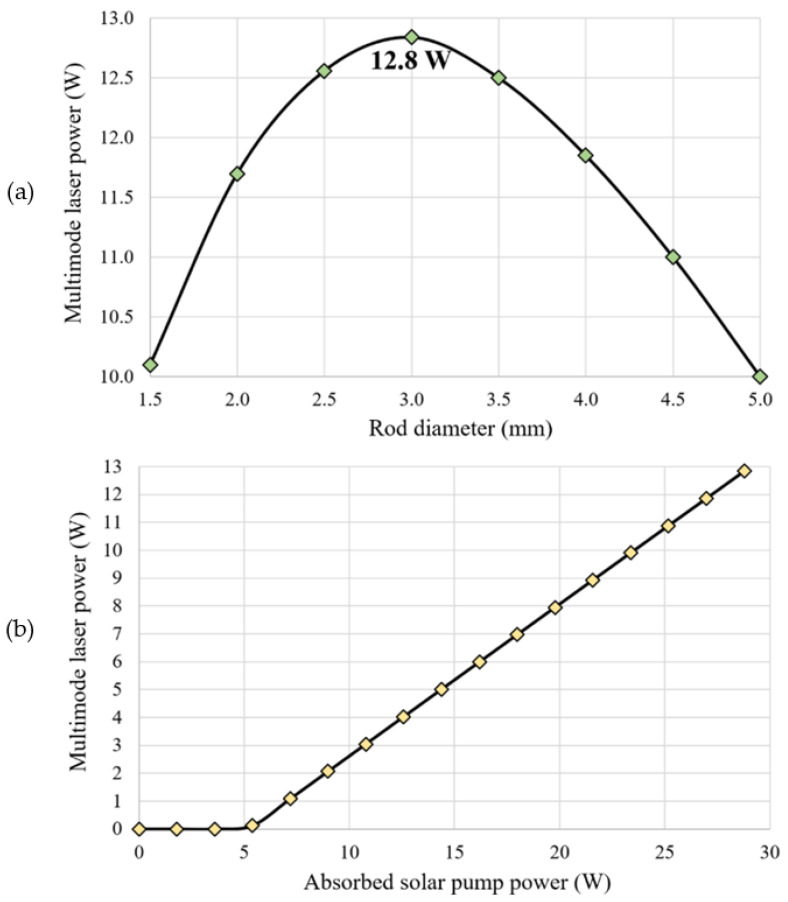
(**a**) Multimode laser power as a function of rod diameter. (**b**) Multimode laser power from one of the four 3.0 mm diameter, 20 mm length Nd:YAG rods as a function of absorbed solar pump power.

All the design parameters of the four-rod solar laser head (conical lens, pump cavity and laser rods) and laser resonator were optimized by ZEMAX^®^ and LASCAD^®^ numerical analysis software, respectively, to achieve the highest multimode laser power from each laser rod. For such, a plane-concave laser resonator with a relatively small *L* was adopted to ensure that the energy of higher-order modes was not wasted by diffraction losses. The multimode laser power from each one of the four rods was numerically studied as a function of the rod diameter (*D_R_*), as represented in Figure 5a. The laser head dimensions, including the rod length (*L_R_*), were optimized for each D_R_. The maximum multimode solar laser extraction of 12.8 W was calculated for each of the four Nd:YAG laser rods with *D_R_* = 3.0 mm and *L_R_* = 20.0 mm for an absorbed solar power of 28.8 W, as presented in Figure 5b. By summing up the multimode laser power calculated from all the four identical laser rods, 51.2 W (4 × 12.8 W) total multimode solar laser power was found, corresponding to 29.0 W/m^2^ collection efficiency and 3.1% solar-to-laser power conversion efficiency.

### 4.2. Tracking Error Compensation Capacity

**Figure 6:**
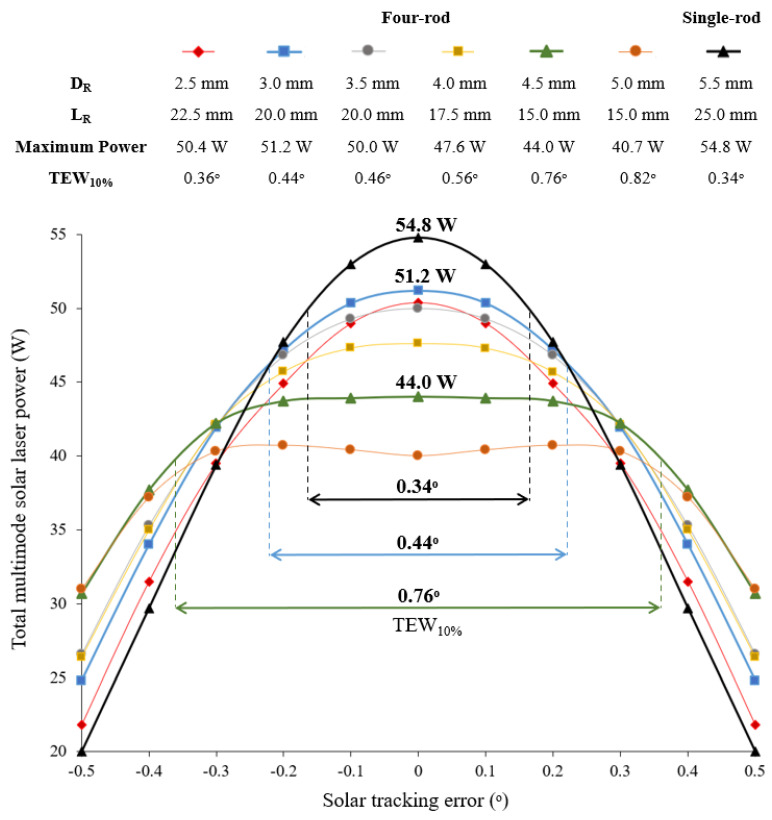
Total multimode solar laser power for the four-rod scheme as a function of the solar tracking error, ranging from the optimal alignment (0.0°) to 0.5° for different rod diameters. A single-rod scheme with *D_R_* = 5.5 mm and *L_R_* = 25.0 mm is also presented for comparison.

The movement of the Earth affects the collected solar radiation on solar systems, and consequently the solar pumping of the laser medium. Solar tracking error shifts the focal spot from its optimal alignment, resulting in less solar laser output power. Solar concentration systems such as point-focusing concentrators need to employ some form of STS with sufficient accuracy to enable higher concentration ratios, radiation flux and thus conversion efficiency [38]. Consequently, it becomes necessary to follow the daily apparent motion of the sun in both altitude and azimuth directions [44]. The solar laser tracking error compensation capacity of the four-rod scheme pumped by the NOVA heliostat-parabolic system was numerically analyzed. Typical solar tracking error up to ±0.5˚ in either azimuthal or altitude directions were considered for the analysis. In Figure 6 is shown the total multimode solar laser power of the four-rod scheme as a function of the solar tracking error in one direction for different *D_R_*. For comparison, a single-rod scheme was also numerically optimized (*D_R_* = 5.5 mm, *L_R_* = 25.0 mm) considering the same pumping conditions (collection area, pumping configuration, active media and resonant cavity). The tracking error width at 10% laser power loss (TEW_10%_) was chosen to analyze the solar laser tracking error compensation capacity.

**Figure 7:**
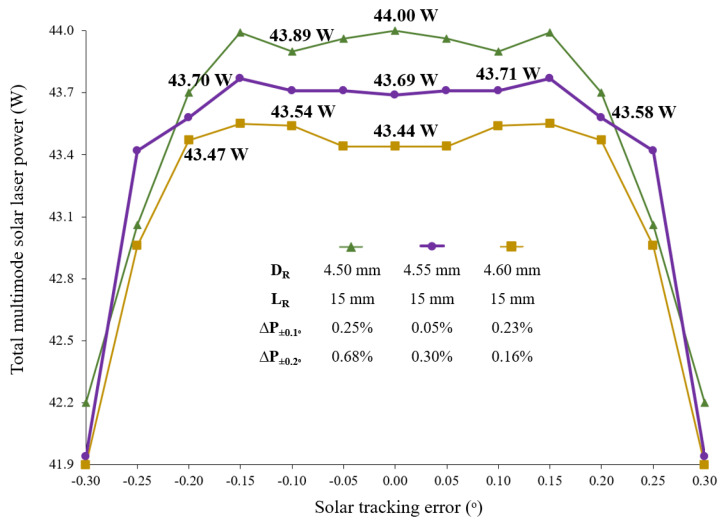
Total multimode solar laser power for the four-rod scheme as a function of the solar tracking error, ranging from the optimal alignment (0.0°) to 0.30° for *D_R_* = 4.50 mm, 4.55 mm and 4.60 mm. The variation in total multimode laser power at ±0.1° solar tracking error (∆*p*_±0.1°_) and ±0.2° solar tracking error (∆*p*_±0.2°_) were presented for each *D_R_*.

The highest multimode solar laser power of 54.8 W was obtained with the single-rod scheme; however, it showed the lowest solar tracking error compensation capacity, with a TEW_10%_ of 0.36°. For the four-rod scheme, the maximum total multimode solar laser power of 51.2 W was calculated with *D_R_* = 3.0 mm and *L_R_* = 20.0 mm. For this case, around 0.44° TEW_10%_ was numerically calculated. As expected, for *D_R_* = 2.5 mm, *L_R_* = 22.5 mm, both total multimode solar power and solar tracking error compensation capacity were lower. Large diameter laser rods significantly improved the tracking error compensation capacity. For *D_R_* = 4.5 mm and *L_R_* = 15.0 mm total multimode solar power of 44.0 W and around 0.76° TEW_10%_ were numerically calculated. Despite the better tracking error compensation capacity calculated for *D_R_* = 5.0 mm, *L_R_* = 15.0 mm, the total multimode solar power was not only lower but also presented less stability with the maximum of 40.7 W found for a solar tracking error of ±0.2°. For an accurate analysis of the solar laser tracking error compensation capacity of the four-rod scheme, the total multimode laser power as a function of the tracking error was analyzed in more detail for the for *D_R_* = 4.50 mm, 4.55 mm and 4.60 mm using a more accurate tracking variation of 0.05°, as shown in Figure 7. The variation in total multimode laser power at ±0.1° solar tracking error (∆*p*_±0.1°_) and ±0.2° solar tracking error (∆*p*_±0.2°_) were calculated as a measure of power stability.

The slightest power variation of only ∆*p*_±0.1°_ = 0.05% was achieved by using four Nd: YAG rods with *D_R_* = 4.55 mm. By considering the tracking error range of ±0.20°, the total multimode laser power calculated with *D_R_* = 4.60 mm showed better stability (∆*p*_±0.2°_ = 0.16%), however its variation at ±0.10° tracking error was higher (∆*p*_±0.1°_ = 0.23%).

**Figure 8:**
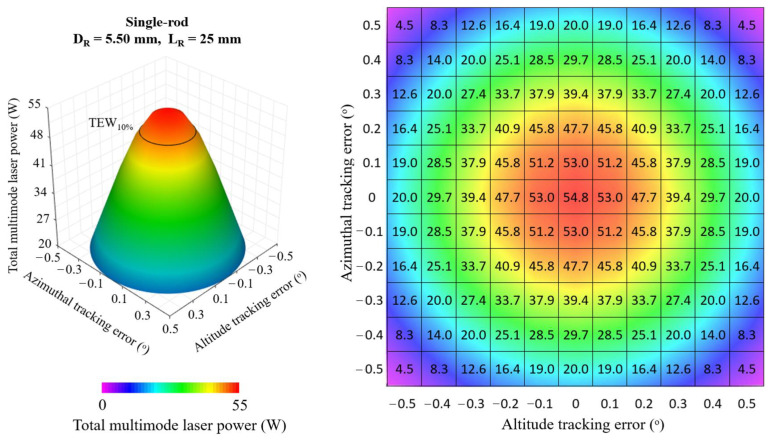
Total multimode solar laser power as a function of the simultaneous tracking error in altitude and azimuthal directions, ranging from the optimal alignment (0.0°) to 0.5°, for the single-rod scheme with *D_R_* = 5.50 mm and *L_R_* = 25 mm.

**Figure 9:**
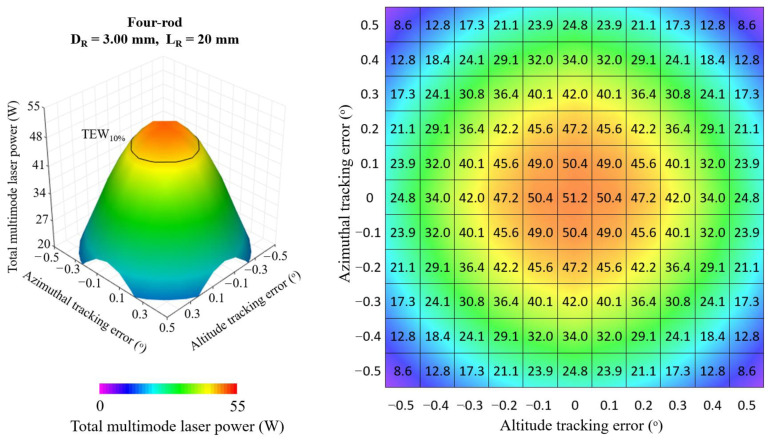
Total multimode solar laser power as a function of the simultaneous tracking error in altitude and azimuthal directions, ranging from the optimal alignment (0.0°) to 0.5°, for the four-rod scheme with *D_R_* = 3.00 mm and *L_R_* = 20 mm.

**Figure 10:**
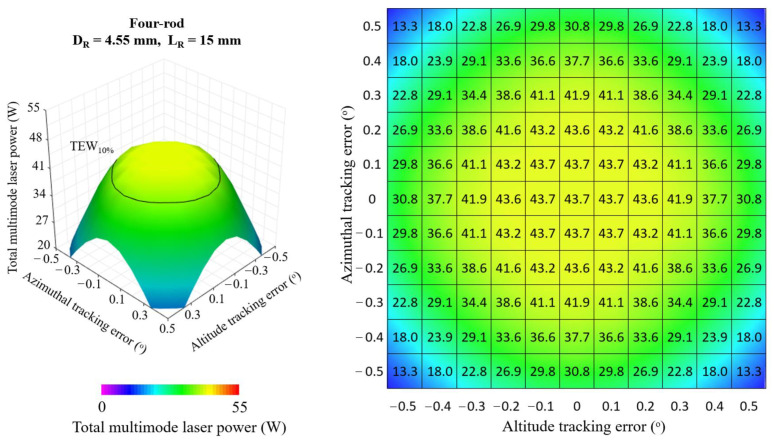
Total multimode solar laser power as a function of the simultaneous tracking error in altitude and azimuthal directions, ranging from the optimal alignment (0.0°) to 0.5°, for the four-rod scheme with *D_R_* = 4.55 mm and *L_R_* = 15 mm.

Figure 8, Figure 9 and Figure 10 show the 3D and 2D top view of the total multimode solar laser powers from the single-rod scheme, the four-rod scheme with *D_R_* = 3.00 mm, *L_R_* = 20 mm and from the four-rod with *D_R_* = 4.55 mm, *L_R_* = 15 mm, respectively, as a function of the altitude and azimuthal solar tracking errors, ranging from 0.0° to 0.5°.

In the optimal alignment (0.0° solar tracking error in both directions), the single-rod scheme (Figure 8) numerically attained the highest multimode solar laser power; however, any slight solar tracking error in either azimuth or altitude axis causes a significant reduction in solar laser output. By using the four-rod scheme (Figure 9 and Figure 10), the concentrated solar power was focused onto the enlarged area comprised of the end faces of the four laser rods, reducing the output laser emission sensitivity to the solar tracking error. This effect was significant, particularly for the four-rod scheme with *D_R_* = 4.55 mm, *L_R_* = 15 mm (Figure 10). The four larger diameter laser rods substantially improved tracking error compensation capacity, enabling the most stable multimode solar power emission in terms of solar tracking error compensation capacity.

**Figure 11:**
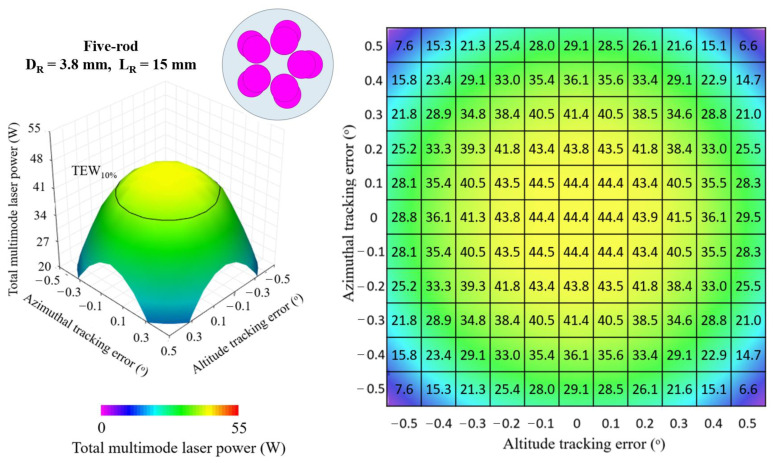
Total multimode solar laser power as a function of the simultaneous tracking error in altitude and azimuthal directions, ranging from the optimal alignment (0.0°) to 0.5°, for the five-rod scheme with *D_R_* = 3.80 mm and *L_R_* = 15 mm.

In order to further study the properties of multi-rod end-pumping configurations on solar tracking error compensation capacity, a five-rod scheme was optimized as in the four-rod case above-described (*D_R_* = 4.55 mm, *L_R_* = 15 mm). Figure 11 shows the 3D and 2D top view of the total multimode solar laser power from the optimized five-rod scheme (*D_R_* = 3.80 mm, *L_R_* = 15 mm) as a function of the altitude and azimuthal solar tracking errors, ranging from 0.0° to 0.5°.

The five-rod scheme (*D_R_* = 3.80 mm, *L_R_* = 15 mm) achieved a maximum total laser power of 44.4 W, leading to 25.1 W/m^2^ collection efficiency and 2.6% solar-to-laser conversion efficiency. Despite slightly outperforming the four-rod scheme (*D_R_* = 4.55 mm and *L_R_* = 15 mm) in multimode solar laser power, the solar tracking error compensation capacity was found reduced, around 0.66° TEW_10%_, 0.05% ∆*p*_±0.1°_ and 1.06% ∆*p*_±0.2°_ were calculated. Moreover, the addition of one extra Nd:YAG rod, and the respective mechanics and resonator mirror, increases the system complexity and cost. Table 1 presents the performance of both the four-rod and the five-rod schemes regarding solar laser efficiencies and solar tracking error compensation capacity.

### 4.3. LASCAD^®^ Solar Laser Thermal Performance Analysis

**Figure 12:**
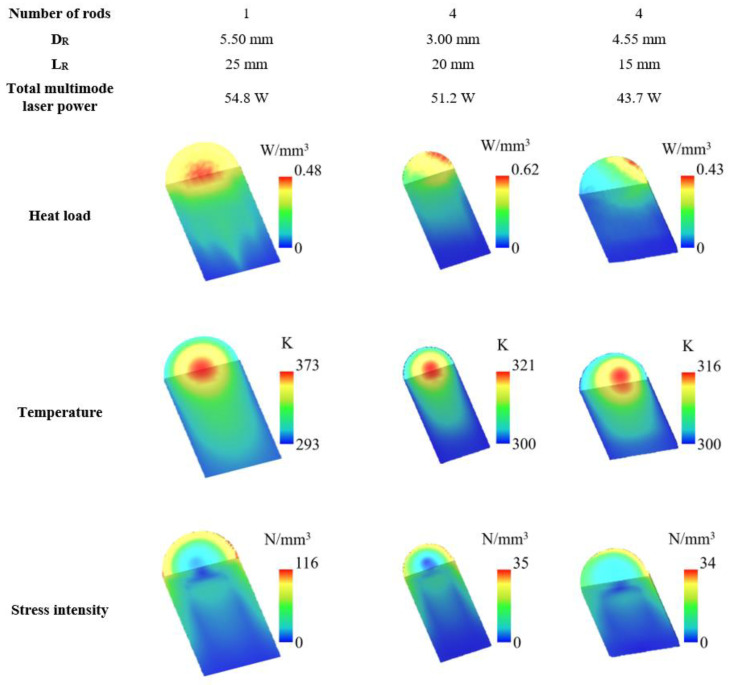
Numerically calculated heat load, temperature and stress intensity distributions of the 5.50 mm diameter, 25 mm length Nd:YAG rod from the single-rod scheme, and one of the four 4.55 mm diameter, 15 mm length and 3.00 mm diameter, 20 mm length Nd:YAG rods from the four-rod scheme that achieved better tracking error compensation capacity and higher total multimode solar laser power, respectively.

The thermal performance of the laser material under solar pumping is of paramount importance in solar-pumped lasers. Therefore, it was also carried out a numerical analysis in order to study the thermal conditions of the Nd:YAG media. In LASCAD^®^ numerical analysis, the absorbed pump flux data from ZEMAX^®^ software were integrated over the Nd:YAG rod volume. The thermal parameters, such as heat load, temperature and stress intensity, were calculated for the single-rod scheme (*D_R_* = 5.50 mm, *L_R_* = 25 mm), and for the four-rod scheme with the rod’s dimensions that achieved better tracking error compensation capacity (*D_R_* = 4.55 mm, *L_R_* = 15 mm) and higher total multimode solar laser power (*D_R_* = 3.00 mm, *L_R_* = 20 mm). The results are summarized in Figure 12, showing a significant reduction in the prejudicial thermal-induced effects in the four-rod scheme.

For an end-side pump scheme, the uneven light flux distribution along the crystal is a serious thermal issue, particularly at the upper-end side of the rod. Despite having the highest total multimode solar laser output power, the 5.5 mm diameter, 25 mm length rod of the single-rod scheme has a more severe stress intensity, reaching a maximum value of 116 N/mm^2^, while by using a four rod-scheme the maximum stress intensity was significantly reduced to 35 N/mm^2^ (*D_R_* = 3.00 mm, *L_R_* = 20 mm) and 34 N/mm^2^ (*D_R_* = 4.55 mm, *L_R_* = 15 mm). Note that for both schemes the maximum stress intensity was lower than the stress fracture limit of 200 N/mm^2^ for Nd:YAG medium [23].

## 5. Discussion

Table 2 presents the recent numerical progress on multi-rod solar laser with Nd:YAG laser rods. The proposed four-rod solar laser head concept was able to produce a maximum of 51.2 W total multimode solar laser power in the numerical analysis, corresponding to 29.0 W/m^2^ collection efficiency and 3.1% solar-to-laser power conversion efficiency, by using four 3.00 mm diameter, 20 mm length Nd:YAG rods. In 2019, Almeida et al. proposed a four-rod scheme able to achieve 59.0 W total multimode solar laser power in the numerical analysis, leading to 19.2 W/m^2^ collection efficiency and 2.0% solar-to-laser conversion efficiency, which is significantly less efficient than the four-rod scheme here proposed [31]. Moreover, the previous four-rod scheme was much more complex, using a quadrangular pyramidal reflector to redirect the focused solar rays towards four laser heads, in comparison to the much simpler single laser head design adopted in the present work. Similar assumptions can be made on the seven-rod scheme proposed by Almeida et al. in 2020 [33]. In this case, the concentrated solar radiation was focused into a single laser head but each one of the seven Nd:YAG rods was pumped within an individual conical cavity, not taking advantage of the shared absorption between rods in the same cavity, and consequently leading to a reduced efficiency. Liang et al. made use of a single conical cavity to pump three and seven Nd:YAG rods [32,35], being able to take advantage of the rods’ shared pumping absorption. The simple three-rod approach numerically obtained 18.6 W total multimode solar laser power by using a heliostat-parabolic system with 1.000 m^2^, being also significantly less efficient than the four-rod scheme here proposed [32]. The seven-rod scheme made use of a lightweight and low-cost Fresnel lens with 4.000 m^2^ effective collection area, obtaining 107 W multimode solar laser power in the numerical analysis. Still, a complex multi-mirror resonator system should be implemented in order to simultaneously extract seven solar laser beams from the single solar laser head [35]. The above-mentioned numerical works have not explored the important issue of solar tracking error compensation capacity, yet it is possible to infer its performance based on the rods’ end face arrangement at the focus. For example, in the three-rod scheme case, the region comprised of the three rods’ end faces does not properly fit the focus, possibly leading to an unstable laser emission regarding solar tracking error compensation capacity. In 2020, Tibúrcio et al. proposed a dual-rod side-pumping solar laser concept for studying its potential on solar tracking error compensation capacity [34]. The concept numerically demonstrated high efficiency when compared to other single-rod side-pumping configurations [47]. Furthermore, great stability was numerically verified, however only along one direction, exhibiting clear asymmetries on the solar tracking error compensation capacity. By using four 4.55 mm diameter, 15 mm length Nd:YAG rods, the four-rod solar laser head concept here proposed significantly enhanced the solar tracking error compensation capacity parameters (TEW_10%_, ∆*p*_±0.1°_, ∆*p*_±0.2°_) when compared to the previous numerical analysis on the side-pumping dual-rod scheme [34].

Table 3 summarizes the most recent progress in multi-rod solar laser performance at the NOVA heliostat-parabolic system, in terms of solar laser collection and solar-to-laser power conversion efficiencies, and tracking error compensation capacity. In order to improve the solar laser tracking error compensation capacity, four 4.55 mm diameter, 15 mm length Nd:YAG rods were adopted in the four-rod solar laser head concept. The concentrated solar power can be directly focused onto the enlarged area comprised of the end face of the four laser rods, enhancing the capture of the sunlight, and consequently leading to a more stable solar laser emission. A total of 0.76° TEW_10%_, 0.05% ∆*p*_±0.1°_ and 0.30% ∆*p*_±0.2°_ were numerically obtained, being 1.27, 74.80 and 21.63 times, respectively, more than the previous experimental record in solar tracking error compensation capacity obtained by using the dual-rod side-pumping horizontal prototype with the same heliostat-parabolic system [36]. Note that Tibúrcio et al. used a side-pumping configuration to pump two Nd:YAG rods, finding a different solar tracking error compensation capacity for the horizontal and vertical setups. However, in this work, the end-side-pumping configuration of the four-rod solar laser head concept presented rotational symmetry, ensuring the same solar tracking error compensation capacity in both directions. Besides the greater stability, the four-rod solar laser head concept achieved 43.7 W total multimode solar laser power, corresponding to 24.7 W/m^2^ collection efficiency and 2.6% solar-to-laser power conversion efficiency, being 1.75 and 1.44 times, respectively, more than those measured with the dual-rod side-pumping prototype [36]. Collection and solar-to-laser conversion efficiencies of the four-rod solar laser head concept were also 1.58 and 1.41 times, respectively, more than the experimental records on multi-rod solar laser efficiencies achieved by Liang et al. [32].

It is worth noting that this four-rod solar laser concept can be applied in the pumping of other solar laser materials, such as Cr:Nd:YAG ceramic medium [26] and Ce:Nd:YAG [48,49], with an absorption spectrum that better fits the solar emission spectrum, leading to higher absorption efficiency, and thus to a further increase in the solar laser power and efficiency. Furthermore, by pumping several thin rods within a single cavity, a very effective rod cooling can be achieved, avoiding the serious thermal issues associated with classical single-thick-rod solar lasers, while allowing the sharing of energy between the thinner rods. However, as demonstrated above by the four-rod versus five-rod analysis, increasing the number of rods does not necessarily lead to better results; moreover, the system becomes inherently more complex and costly, and may be no longer of interest.

## 6. Conclusions

A four-rod single solar laser head concept was proposed using the full collection area of the NOVA heliostat-parabolic system to concentrate the solar radiation towards the laser head. The second-stage conical fused silica lens further concentrated and distributed the solar radiation onto the four Nd:YAG rods. By redistributing the pumping through the four rods, very effective rod cooling was achieved, avoiding the serious thermal lensing and thermal stress issues associated with classical large diameter single laser media. The single conical pump cavity also ensured higher absorption efficiency since solar pump rays not completely absorbed by one of the rods were further absorbed by the other rods, increasing the path length of the rays through the active media, and hence the amount of energy absorbed. Optimum optical pumping and lasing system design parameters were found through ZEMAX^®^ and LASCAD^®^ numerical analysis. Substantial improvement in multimode solar laser performance was numerically achieved with this concept. A maximum total multimode solar laser power of 51.2 W was calculated from the four 3.00 mm diameter, 20 mm length rods, leading to 29.0 W/m^2^ collection efficiency and 3.1% solar-to-laser power conversion efficiency, being 1.58 and 1.41 times, respectively, more than the previous multi-rod experimental records in multimode regime with the NOVA heliostat-parabolic system [32]. Moreover, the solar tracking error compensation capacity was significantly improved. By using four 4.55 mm diameter, 15 mm length Nd:YAG rods, 0.76° TEW_10%_, 0.05% ∆*p*_±0.1°_ and 0.30% ∆*p*_±0.2°_ were numerically calculated, being 1.27, 74.80 and 21.63 times, respectively, more than the previous experimental record in solar tracking error compensation capacity obtained by using the dual-rod side-pumping horizontal prototype with the same heliostat-parabolic system [36]. In addition to the greater stability, the end-side-pumping configuration of the four-rod solar laser head concept (*D_R_* = 4.55 mm, *L_R_* = 15 mm) also enabled an increase of 1.75 and 1.44 times in collection and solar-to-laser power conversion efficiencies, respectively, in relation to that from the experimental the dual-rod side-pumping prototype [36].

Simultaneous solar laser emissions from the four rods could provide new solutions for multi-beam solar-powered lasers applications. Four hollow-core photonic crystal fibres can be used to deliver four solar laser beams or may also be combined by the incoherent beam combining technique to achieve higher solar laser power [52]. Furthermore, the significant improvement in solar tracking error compensation capacity, particularly for a highly efficient end-side-pumping configuration, reduces the need for complex and expensive high-precision solar trackers, leading to a decrease in the cost of solar laser technology.

## Figures and Tables

**Table 1:** Multimode solar laser performance of both the four-rod and the five-rod schemes regarding solar laser efficiencies and solar tracking error compensation capacity.

Parameters	Four-Rod	Five-Rod
Rod dimensions	*D_R_* = 4.55 mm *L_R_* = 15 mm	*D_R_* = 3.8 mm *L_R_* = 15 mm
Total multimode laser power	43.7 W	44.4 W
Multimode laser collection efficiency	24.7 W/m^2^	25.1 W/m^2^
Multimode solar-to-laser conversion efficiency	2.6%	2.6%
TEW_10%_	0.76°	0.66°
∆*p*_±0.1°_	0.05%	0.02%
∆*p*_±0.2°_	0.30%	1.06%
System complexity	Simple	More complex

**Table 2:** Recent numerical progress on multi-rod solar laser performance using Nd:YAG laser rods.

Parameters		Tibúrcio et al., 2020 [34]	Liang et al., 2020 [32]	Almeida et al., 2019 [31]	Almeida et al., 2020 [33]	Liang et al., 2021 [35]	This Work	
							*D_R_* = 3.00 mm *L_R_* = 20 mm	*D_R_* = 4.55 mm *L_R_* = 15 mm
Primary concentrator		Parabolic mirror	Parabolic mirror	Parabolic mirror	Parabolic mirror	Fresnel Lens	Parabolic mirror	
Effective collection area		1.560 m^2^	1.000 m^2^	3.070 m^2^	1.767 m^2^	4.000 m^2^	1.767 m^2^	
Solar irradiance		890 W/m^2^	830 W/m^2^	1000 W/m^2^	950 W/m^2^	950 W/m^2^	950 W/m^2^	
Number of rods		2	3	4	7	7	4	
Pumping configuration		Side-pumping	End-side-pumping	End-side-pumping	End-side-pumping	End-side-pumping	End-side-pumping	
Total multimode laser power		37.7 W	18.6 W	59.0 W	32.2 W	107.0 W	51.2 W	43.7 W
Multimode laser collection efficiency		24.2 W/m^2^	18.6 W/m^2^	19.2 W/m^2^	18.2 W/m^2^	26.8 W/m^2^	29.0 W/m^2^	24.7 W/m^2^
Multimode solar-to-laser conversion efficiency		2.9%	2.2%	2.0%	1.9%	2.8%	3.1%	2.6%
TEW_10%_	Horizontal	0.50°	-	-	-	-	0.44°	0.76°
	Vertical	0.70°						
∆*p*_±0.1°_	Horizontal	2.32%	-	-	-	-	1.59%	0.05%
	Vertical	0.31%						
∆*p*_±0.2°_	Horizontal	11.54%	-	-	-	-	8.47%	0.30%
	Vertical	2.13%						
Characteristics		Efficient for side-pumping	Simple approach	Complex concentration system design	Complex laser head design	Lower cost primary concentrator	Simple approach	
		Asymmetric solar tracking error compensation capacity	Unpromising solar tracking error compensation capacity	No shared absorption	No shared absorption	Complex resonator system	Potential for efficient solar laser emission with uniform solar tracking error compensation capacity	

**Table 3:** Recent experimental progress in multi-rod solar laser performance with the NOVA heliostat-parabolic system and using Nd:YAG laser rods.

Parameters		Tibúrcio et al., 2022 [36] (Experimental)	Liang et al., 2020 [32] (Experimental)	This Work (Numerical)	Improvement over Previous Record (Times)	
				*D_R_* = 4.55 mm *L_R_* = 15 mm		
Effective collection area		1.050 m^2^	1.000 m^2^	1.767 m^2^	-	
Solar irradiance		783 W/m^2^	830 W/m^2^	950 W/m^2^	-	
Number of rods		2	3	4	-	
Total multimode laser power		14.8 W	18.3 W	43.7 W	-	
Multimode laser collection efficiency		14.1 W/m^2^	18.3 W/m^2^	24.7 W/m^2^	1.75 [36]	1.58 [32]
Multimode solar-to-laser conversion efficiency		1.8%	2.2%	2.6%	1.44 [36]	1.41 [32]
TEW_10%_	Horizontal	0.60°	-	0.76°	1.27 [36]	
	Vertical	1.40°			0.54 [36]	
∆*p*_±0.1°_	Horizontal	3.74%	-	0.05%	74.80 [36]	
	Vertical	0.75%			15.00 [36]	
∆*p*_±0.2°_	Horizontal	6.49%	-	0.30%	21.63 [36]	
	Vertical	2.63%			8.77 [36]	

## Data Availability

Not applicable.
